# Design of exceptionally strong and conductive Cu alloys beyond the conventional speculation via the interfacial energy-controlled dispersion of γ-Al_2_O_3_ nanoparticles

**DOI:** 10.1038/srep17364

**Published:** 2015-11-30

**Authors:** Seung Zeon Han, Kwang Ho Kim, Joonhee Kang, Hongrae Joh, Sang Min Kim, Jee Hyuk Ahn, Jehyun Lee, Sung Hwan Lim, Byungchan Han

**Affiliations:** 1 Structural Materials Division, Korea Institute of Materials Science, Changwon, 51508, Korea; 2 School of Materials Science and Engineering, Pusan National University, Pusan, 46241, Korea; 3 Department of Energy Systems Engineering, DGIST, Daegu, 42997, Korea; 4 Research and Development Division, KOS Ltd., Yangsan, 50592, Korea; 5 Research and Development Division, Seowon, Ansan, 15599, Korea; 6 Department of Materials Science and Engineering, Changwon National University, Changwon, 51140, Korea; 7 Department of Advanced Materials Science & Engineering, Kangwon National University, Chuncheon, 24341, Korea; 8 Department of Chemical and Biomolecular Engineering, Yonsei University, Seoul, 03722, Korea

## Abstract

The development of Cu-based alloys with high-mechanical properties (strength, ductility) and electrical conductivity plays a key role over a wide range of industrial applications. Successful design of the materials, however, has been rare due to the improvement of mutually exclusive properties as conventionally speculated. In this paper, we demonstrate that these contradictory material properties can be improved simultaneously if the interfacial energies of heterogeneous interfaces are carefully controlled. We uniformly disperse γ-Al_2_O_3_ nanoparticles over Cu matrix, and then we controlled atomic level morphology of the interface γ-Al_2_O_3_//Cu by adding Ti solutes. It is shown that the Ti dramatically drives the interfacial phase transformation from very irregular to homogeneous spherical morphologies resulting in substantial enhancement of the mechanical property of Cu matrix. Furthermore, the Ti removes impurities (O and Al) in the Cu matrix by forming oxides leading to recovery of the electrical conductivity of pure Cu. We validate experimental results using TEM and EDX combined with first-principles density functional theory (DFT) calculations, which all consistently poise that our materials are suitable for industrial applications.

Nanoscale materials have demonstrated novel properties that deviate from the traditional laws for bulk materials. Examples include reddish-colored gold^1^, mechanically strong nanostructured metals^2^, transparent magnets^3^ and superconductors^4^. The design of these materials involves tuning one of the four inherent features: optical, mechanical, magnetic and electrical properties.

Multifunctional devices are important to meeting various human requirements and the environmental complexity of operational conditions. Considering that component materials of any device play a key role in determining the overall efficiency successful design of the multifunctional systems requires a fundamental understanding of the origin of material properties and sound integration of the individual material into practical engineering applications such as the semiconductor and automobile industries.

However, the design of materials with multivariate functionality is strictly limited by the conventional laws, especially when the desired properties appear to be mutually exclusive. For example, enhancing the mechanical strength of a Cu alloy without sacrificing the electrical conductivity is a long-standing example of the issue. Traditionally, the strengthening mechanical properties of metallic alloys was based on the complicated manipulation of the lattice structure of the parent material, which inevitably manipulates or disrupts electron transport in the desired direction reducing electric conductivity and often decreases ductility^5^,^6^,^7^. Two widely employed methods^8^,^9^,^10^,^11^,^12^,^13^ utilize either the modification of grain structures or the addition of foreign elements followed by heat treatments.

In this paper, we demonstrate Cu alloys with outstanding mechanical properties and electrical conductivity beyond conventional restrictions. Our objective is to simultaneously improve the mechanical strength and electrical conductivity, which are mutually in trade-off nature. Over a bulk Cu matrix, we designed hybrid interface structures by uniformly dispersing Al_2_O_3_ via internal oxidation process using externally supplied oxygen. The mechanical strength of Cu was improved through a dispersion-hardening mechanism driven by the nucleation and growth processes of the nano-scale oxide particles. We recovered the electrical conductivity of Cu that was degraded by residual O (remains inside the Cu matrix from the stoichiometric relationship between Al and O) through addition of Ti. Our results indicated that Ti forms various oxides such as TiO_2_, TiO and ternary phases with Al and O, leaving minimal impurities inside the Cu matrix.

Similar to our study there have been endeavors to disperse Al_2_O_3_ particles in a Cu matrix. Especially it was reported that the mixture of Ti oxide with Al_2_O_3_ show the possibility of decreasing oxide particle size in copper matrix^14^,^15^ introducing mixture of various oxide in copper matrix. However it has been known as difficult to obtain uniform distribution of the particles^16^,^17^. For example, it was reported that solid-state mixtures of Ti with Al_2_O_3_ might decrease the oxide particle sizes in a copper matrix^17^. Differently from the previous work the key step we made in this study was to oxidize Al at high temperature (T = 980 °C) via an internal oxidation process and to control the interface energies between the oxides and the Cu matrix with extra-doped Ti. We observed that the oxide particles completely phase transformed from irregular to spherical-type morphologies by dissolving the Ti, resulting in the uniform dispersion of the Al_2_O_3_ nanoparticles in the Cu matrix.

Differently from the previous works our materials are ternary solid solutions of Cu-Al-Ti not just solid-state mixtures of Cu-Al with various metals (Ti, Zr, Hf and so on. Therefore, Ti is solvated substitutionally in Cu and Al lattices. To accomplish the material design of the ternary solid solution we integrated the advanced fabrication technologies and the structural characterization on an atomic resolution and the first principles calculations. The systematic methodologies enabled us to observe new structural phases of Ti-Al-O oxides and Ti-doped Al_2_O_3_ nanoparticles, which were missing in the experiments with the solid-state mixture. More interestingly, we identified that the morphologies of those phases in the ternary solid solutions are sensitive to interfacial energies, which is, in turn, controlled by amounts of doped Ti. We confirmed that our materials could be processed at high temperature (T = 950 °C) while keeping the oxide particle sizes smaller than about 10 nm improving both mechanical strength and electrical conductivity substantially.

We chose Ti on the ground that it has a higher melting temperature^18^ than Cu easing the heat treatment processes and its solubility in the Cu matrix is as high as in Al enabling to the formation of solid solutions with both Cu and Al. Thus, it is possible to solvate Ti into Al_2_O_3_ during the internal oxidation process to regulate the interfacial energies and the impurity level in Cu. We found that the mechanical properties and electrical conductivity of Cu were enhanced by the spheroidizing of Al_2_O_3_ nanoparticles with the Ti solutes. The results were validated using high-resolution transmission electron microscopy (HRTEM) observations and first-principles density functional theory (DFT) calculations.

## Experimental procedure

**Figure 1:**
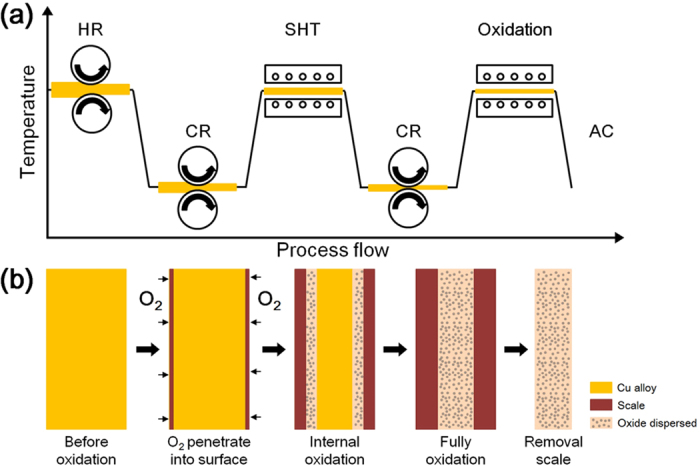
Schematic flow diagram showing the process in (**a**) and in (**b**) the internal oxidation method to design Al_2_O_3_ nanoparticle dispersed Cu alloys.

The 99.9 % pure Cu, Al and Ti were used to prepare Cu-Al and Cu-Al-Ti alloy ingots by atmospheric induction melting and permanent mold casting. We designed four different Cu alloys as a function of the relative composition of Al/Ti and with/without internal oxidation (Table 1). The ingots were hot rolled in the uniaxial direction at 980 °C after being held for one hour. The surface of each plate was scalped to an approximate thickness of 0.5 mm and cold rolled to a 75 % thickness reduction. The sheets of the cold-rolled plates were oxidized at 980 °C for two or four hours at ambient atmosphere. Oxides (Cu_2_O) on the oxidized sheets was removed by wire brushing and ultrasonic cleaning. Figure 1 depicts a schematic flow diagram of the overall fabrication of the oxide-dispersed Cu alloy composite. The wire-type specimen was fabricated by drawing at room temperature after oxidation at 980 °C for 1 hour.

The Vickers hardness was measured on a 100 g load using a hardness tester (FM-700, Future Tech. Corp.). The electrical conductivity was evaluated by the double-bridge method (portable double bridge 2769, Yokogawa M&C). Tensile strength was measured using a tensile tester (EZ-L, Shimadzu) for plate or wire-type specimens.

Characterization of alumina was performed using a 200 kV field-emission transmission electron microscopy (TEM, JEOL, model: JEOL-2100F) equipped with an energy-dispersive X-ray spectroscopy (EDS) detector and a scanning TEM. The TEM specimens were prepared by jet polishing with a Tenupol-5 (Struers) in an etchant solution composed of 250 ml phosphoric acid, 500 ml distilled water, 250 ml ethanol, 50 ml propanol, and 5 g urea (D2). The volume density of the particles was measured with assuming the thickness of the specimen as approximately 50 nm. The TEM samples were fabricated using the standard carbon extraction replica method for HRTEM analysis of the chemical compositions and atomic structures of the nanoparticles. We performed the carbon extraction replica method by mechanical polishing in an aqueous solution containing 0.5 ml diamond colloidal particles. The samples were then collected on a carbon film using a 3 % nital solution chemical attack^19^.

We performed DFT calculations using Vienna *Ab-initio* Simulation Package (VASP)^20^ with the Projector Augmented Wave (PAW)^21^ pseudo-potentials and the Perdew-Burke-Ernzerhof (PBE)^22^ exchange-correlation functional. A plane wave cutoff of 400 eV was used for all calculations. We integrated the Brillouin zone with a gamma point scheme of 3 × 1 × 1, 3 × 1 × 1, and 3 × 3 × 1 k-points for interface model systems based on γ-Al_2_O_3_(100), γ-Al_2_O_3_(110), and γ-Al_2_O_3_(111), respectively. The γ-Al_2_O_3_ was based on Pinto’s model system^23^.

## Results and Discussion

**Figure 2:**
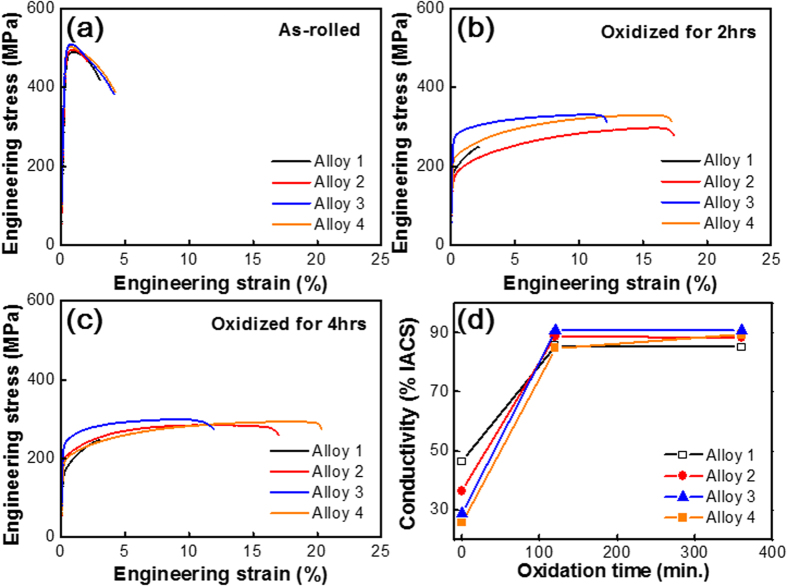
Stress vs. strain relationships of Cu-Al and Cu-Al-Ti alloys before internal oxidation in (**a**), and in (**b,c**) after 2 and 4 hours of oxidations at 980 °C in ambient atmosphere, respectively. Electric conductivity of Cu alloys with varying oxidation time was shown in (**d**).

Figure 2 illustrates the stress versus strain behavior and the electrical conductivities of the four Cu alloys before and after the internal oxidation. All samples for the tensile tests were designed according to ASTM-E8M standard.

Our results indicate that the mechanical properties of all four Cu alloys are sensitive to Ti composition and internal oxidation. Figure 2a demonstrates that, without the internal oxidation mechanical properties, all four Cu alloys are almost identical regardless of Ti composition. The internal oxidation modifies the mechanical behaviors of the Cu alloys, as depicted in Fig. 2b,c. Cu alloys with more Ti resulted in higher yield, greater tensile strength and improved mechanical ductility. The underlying mechanism is at the reduced and homogenized interfacial energies of our ternary solid solution, which is further controlled by the amounts of doped Ti.

Figure 2d clearly shows that the electrical conductivity of a Cu alloy was improved by two hours of internal oxidation. Longer oxidations demonstrated saturation. Alloy 3, which contained equal amounts (40 wt.%) of Ti and Al, demonstrated the highest electrical conductivity (above 90 % of the IACS value).

Without the internal oxidation, Cu alloys with a higher composition of Ti or Al resulted in lower electrical conductivity. As previously reported, Ti degrades more than Al^24^. After internal oxidation, both Ti and Al enhance the electrical conductivity of the Cu alloy. The underlying mechanism may be at the formation of various binary (Ti-O) and ternary (Ti-Al-O) oxides. These oxides remove impurities in the Cu matrix and recover the electrical conductivity of pure Cu.

**Figure 3:**
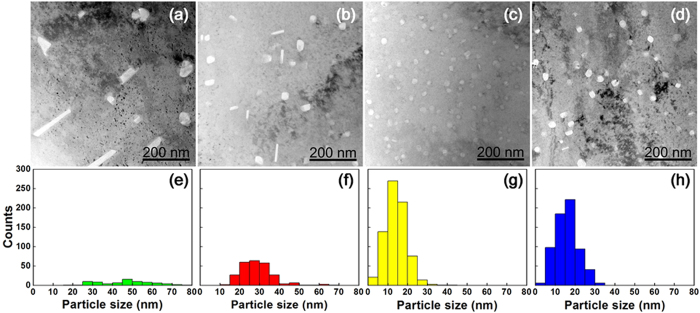
TEM images of the microstructures for Cu-based alloys of Cu-0.8%Al in (**a**) and in (**b**) Cu-0.7%Al-0.1%Ti, for (**c**) Cu-0.4%Al-0.4%Ti and in (**d**) Cu-0.6%Al-0.4%Ti. All materials were oxidized for 2 hours at 980 °C in 1 atmosphere. Distribution of the dispersed oxide nanoparticles in each alloy was plot as a function of size at (**e**–**h**), respectively.

Figure 3 presents the TEM observations of the Al_2_O_3_ nanoparticles dispersed over the four Cu alloys after two hours of internal oxidations at 980 °C and 1 atm. These images show that the morphologies and spatial distribution of the dispersed Al_2_O_3_ are sensitive to Ti composition. Increasing the relative weight fraction of Ti to Al decreases the oxide particle size and increases the total number of particles.

The morphology of the oxide particle transforms from planar (or irregular) to uniform spherical. This ‘spheroidizing’ phenomenon was observed primarily for metal alloys^8^,^9^,^10^,^11^ but is rarely reported for coherent or semi-coherent interfaces between oxide particles and metals. This structural change suggests that added Ti is critical to decreasing and homogenizing the interfacial energies between the Cu matrix and the dispersed Al_2_O_3_ nanoparticles.

**Figure 4:**
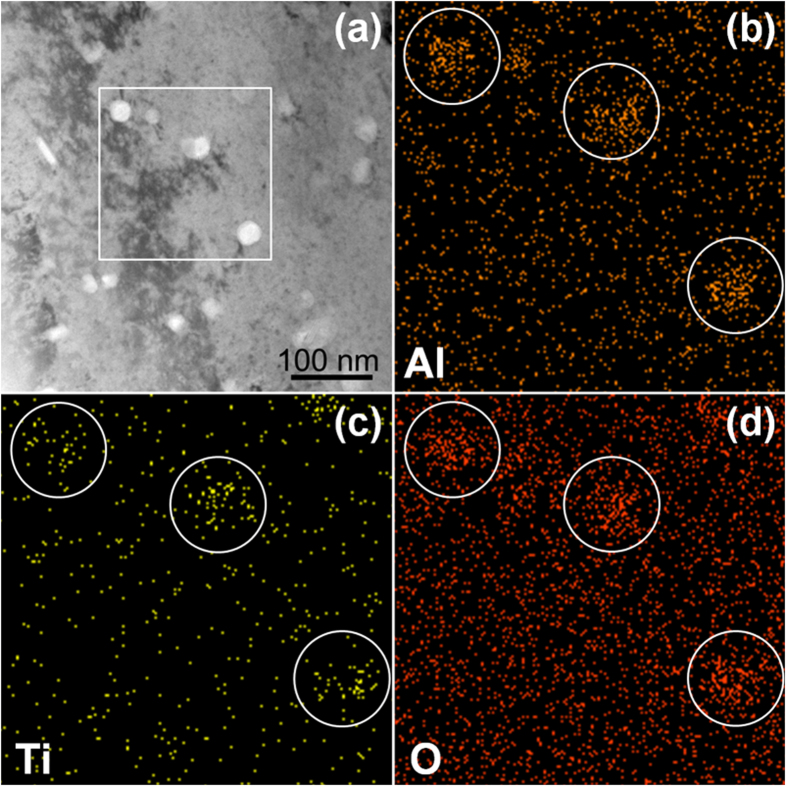
EDX analysis of dispersed γ-Al_2_O_3_ nanoparticles in Cu-0.6%Al-0.4%Ti.

**Figure 5:**
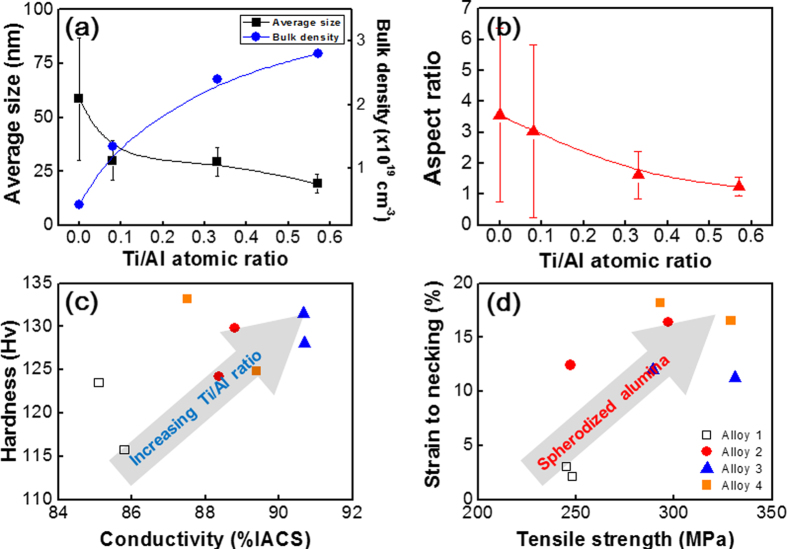
Averaged sizes and volume densities in (**a**) and in (**b**) aspect ratios of dispersed oxide nanoparticles in Cu matrix after 2 hours of oxidation at 980 °C under ambient atmosphere. Relationships of conductivity-hardness and strength-ductility for the oxide nanoparticles after oxidations for 2 or 4 hours were shown at (**c**,**d**), respectively.

To characterize the detailed compositions of the oxides, we used energy-dispersive X-ray (EDX) spectroscopic analysis for Alloy 4 in Table 1 (Cu-0.6Al-0.4Ti). The results (Fig. 4) indicated that Ti partially substitutes Al in Al_2_O_3_ nanoparticles (i.e., a solid solution). Figure 5 illustrates the average particle size, aspect ratio and volume density of the oxide particle over the Cu alloys as a function of the *atomic* fraction of Ti/Al. This figure provides clear correlations between these parameters. The particle size and the aspect ratio decrease as the atomic fraction increases. The volume density is proportional to the atomic fraction. Alloy 4 contains more Ti and Al (in terms of total *weight*) than Alloy 3, and both the size and the aspect ratio are larger, but the volume density is almost identical. Therefore, atomic fractions of Ti to Al describe the system better than weight.

Alloys 1 and 4 have almost the identical amount of the 2^nd^ element in atomic fraction, but the mechanical properties and electrical conductivity are different after the internal oxidation process (Fig. 2). The internal oxidation process, with the appropriate amount of Ti in the Al_2_O_3_ nanoparticles, is critical in controlling the interfacial energies. There is a theory describing the strengthening mechanism^25^,^26^,^27^,^28^ (uniformly dispersed oxide particles with spherical morphology improve the mechanical strength of an alloy matrix for a given particle volume). The increased mechanical strength is due to the impeded dislocation movement. The spheroidized oxide particles also enhance mechanical ductility by removing the stress concentrated at the interfaces between the oxides and the alloy matrix.

**Figure 6:**
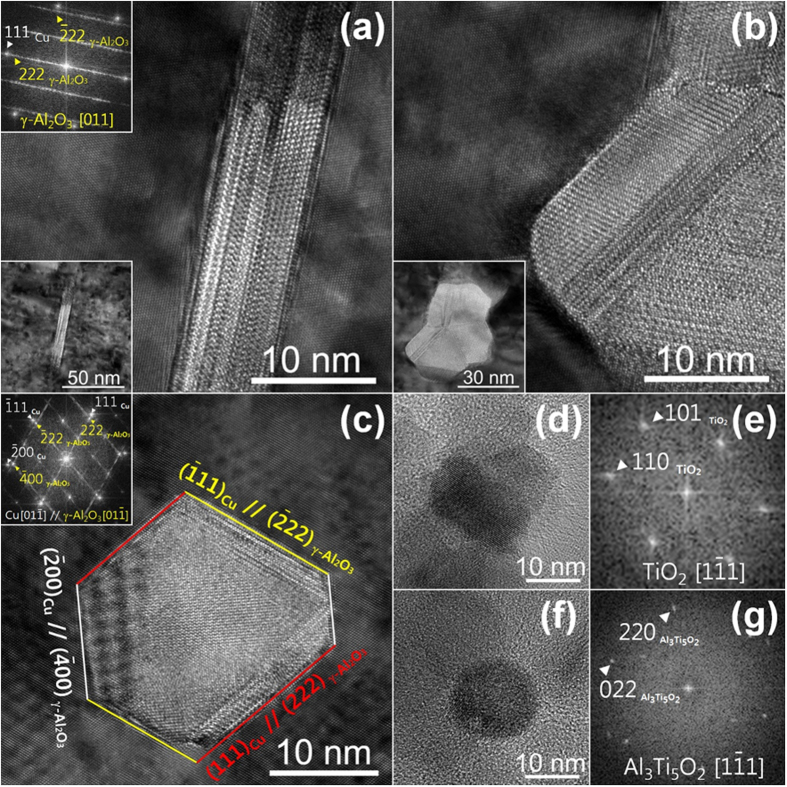
HRTEM images of dispersed γ-Al_2_O_3_ nanoparticles in Cu-0.8%Al alloy of planar in (**a**) and rectangular shapes in (**b**). Images in (**c**) represent morphology of Ti soluted *γ*-Al_2_O_3_. The images (**d,e**) are for TiO_2_, while (**f,g**) are for Al_3_Ti_5_O_2_ nanoparticles in Cu-0.4%Al-0.4%Ti alloy after internal oxidation. The (**d**)~(**g**) were observed in replica.

Figure 6 illustrates the HRTEM analysis of the pure Al_2_O_3_ nanoparticles dispersed in Alloy 1 and Alloy 3 with Ti solutes inside (Ti-Al_2_O_3_) after two hours of internal oxidation at 980 °C and 1 atm. Alloy 1 includes only gamma-phase alumina (γ–Al_2_O_3_, face-centered cubic) with planar and irregular morphologies. This alloy was originated from the interface energies between the Cu matrix and the dispersed γ–Al_2_O_3_ nanoparticles. Our HRTEM analysis observed only 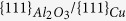 interfaces, which are most likely the planes of the lowest interfacial energy and force γ–Al_2_O_3_ to grow faster in only one direction. The dispersed Ti-Al_2_O_3_ nanoparticles in Alloy 3 appeared as polyhedrons (Fig. 6c). HRTEM measurements revealed additional interfaces of 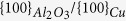 and 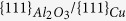. These findings represent the Ti mitigated interfacial energy differences between the dispersed γ–Al_2_O_3_ and the Cu matrix. The dispersed γ–Al_2_O_3_ in Alloy 3 grew into spherical structures that were smaller than the oxide nanoparticles in Alloy 1.

Al_2_O_3_ and Ti should not form a solid solution at ambient conditions according to the thermodynamic phase diagram^29^. However, these materials do form oxides by reacting with O over the Cu matrix. This reaction allows Ti to partially replace Al and create the Ti-soluted γ–Al_2_O_3_. Other oxides formed in Alloy 3, such as TiO_2_ and Al_3_TiO_2_, were much smaller than the Ti-soluted γ–Al_2_O_3_. However, these oxides may increase conductivity because the oxide formation further decreased the solute in the Cu matrix.

Figure 5c,d illustrate the mechanical properties (strength, hardness and ductility) and electrical conductivities of the four Cu alloy systems after internal oxidation. The two material properties, mechanical strength and electrical conductivity, were speculated as mutually exclusive. Figure 2, however, shows that we overcame the conventional limit by designing hybrid interfaces between dispersed γ–Al_2_O_3_ nanoparticles and the Cu alloy matrix with the addition of controlled amounts of Ti solutes. Alloy 3 demonstrated 7 % enhanced electrical conductivity and 300 % enhanced mechanical ductility compared to Alloy 1. The fundamental mechanism of Alloy 3 was the spheroidizing of the dispersed Al_2_O_3_ nanoparticles by Ti solute through homogenized interfacial energies with the Cu matrix.

**Figure 7:**
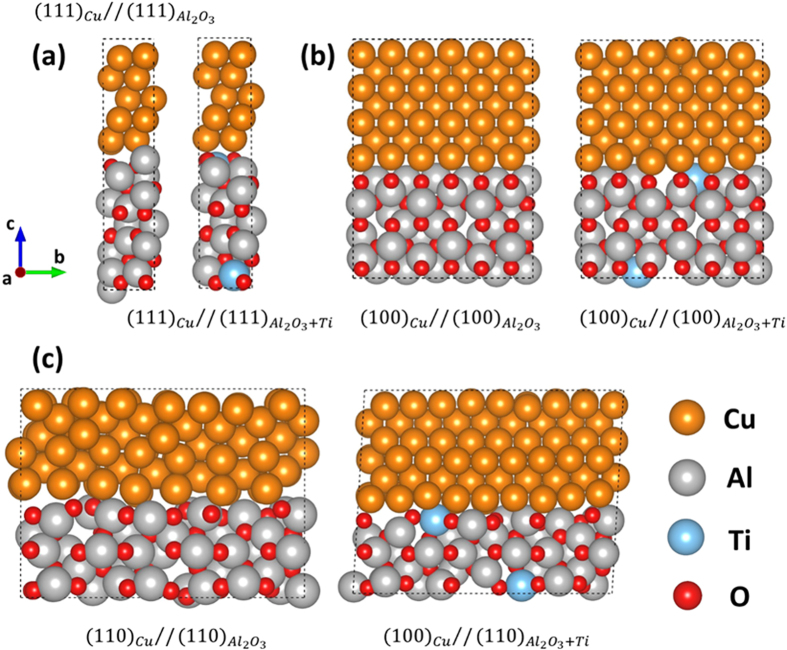
Thermodynamically stable interface structures for the Cu matrix and γ-Al_2_O_3_ nanoparticles with and without Ti solutes captured by first principles DFT calculations. In (**a**) Cu(111)/Al_2_O_3_(111), Cu(111)/Al_2_O_3_ + Ti(111), (**b**) Cu(100)/Al_2_O_3_(100), Cu(100)/Al_2_O_3_ + Ti(100), and (**c**) Cu(110)/Al_2_O_3_(110), Cu(100)/Al_2_O_3_ + Ti(110).

Using first-principles DFT calculations, we validated the experimental observations of the multifunctionality of Cu alloys dispersed with Ti-soluted γ–Al_2_O_3_ nanoparticles. We created model systems of γ–Al_2_O_3_//Cu to simulate interface structures of (100), (110) and (111) facets (Fig. 7). Table 2 provides our DFT results demonstrating that Ti thermodynamically prefers to partially substitute for Al in the (100)γ–Al_2_O_3_//(100)Cu and the (110)γ–Al_2_O_3_//(110)Cu but not in the (111)γ–Al_2_O_3_//(111)Cu interfaces. To evaluate the thermodynamic stability of each structure, we calculated the interfacial decohesion energy, *W*_*de*_, defined in Eq. (1):


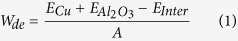


where 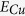, 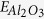, and 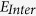 are the energy of Cu, Al_2_O_3_ and the total interface system of Al_2_O_3_//Cu, respectively (calculated by the DFT method). 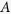 represents the interfacial area between γ–Al_2_O_3_ and the Cu matrix. Increased positive 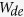 is correlated with increased thermodynamic stability. 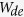 values (DFT calculations) are reported in Table 3. Our results indicate that without Ti, the (111)γ–Al_2_O_3_//(111)Cu interface is the most stable. This finding supports our experimental results (Alloy 1). Ti doping the 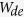 for the (100)γ–Al_2_O_3_//(100)Cu interface structure was substantially increased, whereas (111)γ–Al_2_O_3_// (111)Cu was decreased. Therefore, the interfacial energies for the different facets of the γ–Al_2_O_3_//Cu were homogenized by adding Ti as suggested by the HRTEM observations.

**Figure 8:**
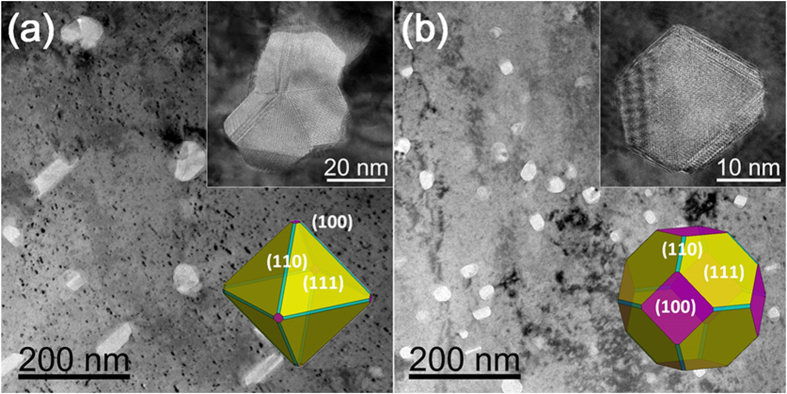
TEM images of dispersed alumina nanoparticles at Cu-8Al in (**a**) and in (**b**) at Cu-4Al-4Ti alloys after internal oxidations. The insets represent the particle structures predicted by Wulff construction method based on ab-initio calculated interface energies.

Using the DFT calculated interfacial energies, we predicted the morphologies of dispersed γ–Al_2_O_3_ nanoparticles with and without added Ti using the Wulff construction method^30^. Figure 8 shows both the images observed by TEM and the DFT calculations (insets). Our first-principles DFT calculations support the underlying mechanism for the high mechanical strength, ductility and electrical conductivity of Cu alloys.

**Figure 9:**
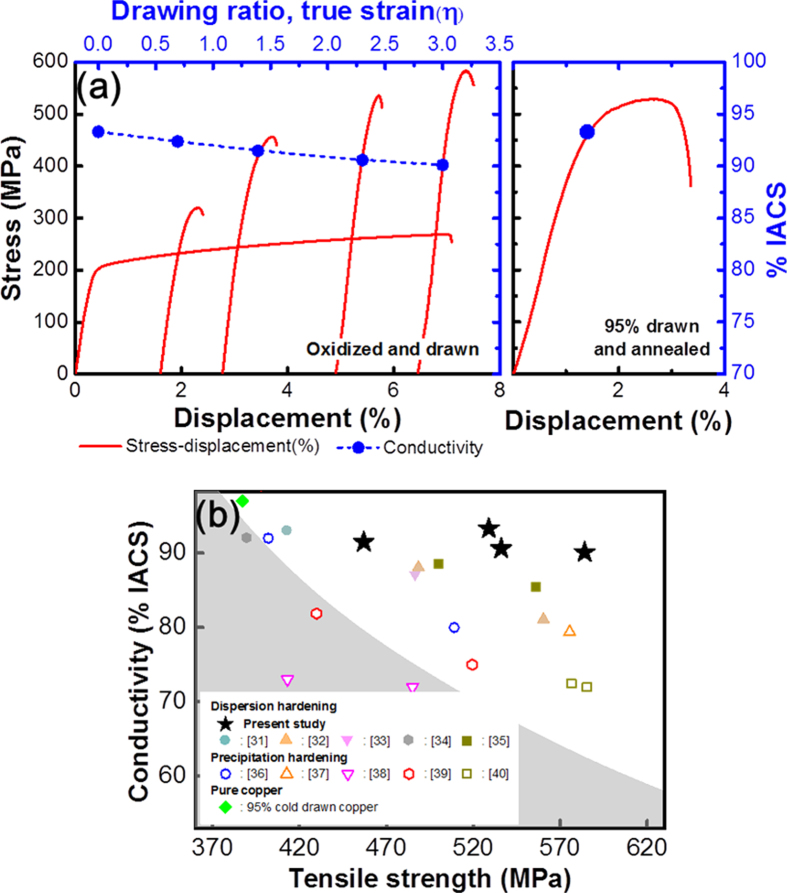
Plot in (**a**) represents electric conductivity and mechanical strength of Cu-0.6%Al-0.4%Ti alloy as function of drawing ratio and successive annealing process, and in (**b**) comparison of our Cu alloys in electric conductivity and tensile strength with previously reported materials.

To determine whether our materials are appropriate for industrial applications, we processed Alloy 4 into a wire of 0.95 mm in diameter followed by the internal oxidation process at 980 °C for one hour. After removal of the oxide scales on the Cu surface, the diameter decreased to 0.63 mm. We further reduced the cross-sectional area of the wire to 5 % of the initial value with a room-temperature drawing process. Figure 9a reports the measured tensile strength and the electrical conductivity of the drawn wire as a function of drawing ratio (true strain *η* = *ln*(A_0_/A), where A_0_ and A are the cross-sectional area of the wire before and after drawing, respectively. The electrical conductivity and the tensile strength of the oxidized wire were measured as 93.32 % IACS and 269 MPa, respectively (Fig. 9a). The slight deviation in the electrical conductivity from the IACS value can be attributed to the geometry of the wire specimen. The electrical conductivity measured for a wire sample is more relevant than a plate-type structure because the cross-sectional area and length of the wire can be precisely defined at any stage of the testing procedure.

Our results indicated that the tensile strength was increased by the work hardening and the electrical conductivity of the wire slightly decreased (3 % from the initial value of Alloy 4) despite the fact that it was processed with a high drawing ratio (true strain *η* = 3). We demonstrated that the wire fabricated from Alloy 4 could be work hardened at room temperature and function in industrial applications.

We applied the thermomechanical process to our Cu alloy with 95 % drawing and annealing at 160 °C for 30 minutes. Surprisingly, the mechanical strength of the material decreased only less than 10 %. Furthermore, as shown in stress-displacement curve (Fig. 9a) the region of uniform deformation spans about 2 %. Another important thing to note is that electrical conductivity was improved as high as 93 % IACS with mechanical strength of 530 MPa and 2.7 % ductility even after the thermomechanical treatment. These results can be ascribed to the high thermal stability of the Cu alloy strengthened by the dispersed oxide particles.

Compared to previously reported copper alloys^31^,^32^,^33^,^34^,^35^,^36^,^37^,^38^,^39^,^40^, our materials show much better performances in simultaneous mechanical strength and electric conductivity. For example, with respect to Cu-Al^32^ alloy the two functionalities of our materials have weaker to slightly stronger ultimate tensile strength values (depending on the processing conditions) of 450–584 versus 550–560 MPa, and significantly higher conductivities (90 % versus ~81% IACS) for all conditions for the similar volume fraction (Fig. 9b). Figure 9 illustrates that there is room for further enhancement of the mechanical strength. We report exceptional performance of our specimen in well-established processes in alloy manufacturing. Our study may be an important step toward improving mechanical strength by combining powder metallurgy or grain boundary engineering technology with severe plastic deformation^39^,^41^,^42^.

## Conclusion

We designed strong, ductile, and conductive Cu alloys by uniformly dispersing spherical structures of γ–Al_2_O_3_ nanoparticles using a decreased and controlled total interface energy with added Ti solid solutes. Although these materials were previously well established in the industrial sector, the multifunctionality we observed was beyond conventional expectations. The critical mechanism originated from the homogenized interfacial energies of the γ–Al_2_O_3_//Cu in the Ti solution to primarily oxide particles and the effective removal of impurities inside the Cu matrix (thermodynamic formations of various oxides with Ti). Nevertheless, we showed the ductility of our materials can be further enhanced by appropriate thermomechanical treatments: for instance, the recovery and recrystallization process for a severely plastically deformed material. And just scaling up of our specimen size probably improves the ductility level as well. We propose the fabrication of a competitive copper alloy can be combined with post-fabrication methods, such as powder metallurgy or the inclusion of nanoscale grains in alloy matrix. This method may result in excellent mechanical properties and electrical conductivity.

## Additional Information

**How to cite this article**: Han, S. Z. *et al.* Design of exceptionally strong and conductive Cu alloys beyond the conventional speculation via the interfacial energy-controlled dispersion of γ-Al_2_O_3_ nanoparticles. *Sci. Rep.* **5**, 17364; doi: 10.1038/srep17364 (2015).

## Figures and Tables

**Table 1:** Four Cu alloys with varying Al and Ti compositions.

Composition (wt%)	Cu	Al	Ti	Ti/Al (wt%)	Ti/Al (at%)
Cu-0.8Al	Bal.	0.8	–	0	0
Cu-0.7Al-0.1Ti	Bal.	0.7	0.1	14	8
Cu-0.4Al-0.4Ti	Bal.	0.4	0.4	100	57
Cu-0.6Al-0.4Ti	Bal.	0.6	0.4	58	33

**Table 2:** DFT calculated Ti doing energy in the hybrid interface structures of Cu and the dispersed γ-Al_2_O_3_ nanoparticles.

	Al_2_O_3_ (100) (eV/Å^2^)	Al_2_O_3_ (110) (eV/Å^2^)	Al_2_O_3_ (111) (eV/Å^2^)
Cu (100)	0.089	0.042	0.283
Cu (110)	0.088	0.051	−
Cu (111)	0.084	0.045	0.310

**Table 3:** DFT calculated interface decohesion energies between Cu matrix and dispersed Al_2_O_3_ particles with and without Ti solute in the oxides.

(J/m^2^)	Al_2_O_3_ (100)	Al_2_O_3_ (100)Ti doped	Al_2_O_3_ (110)	Al_2_O_3_ (110)	Al_2_O_3_ (111) Ti doped	Al_2_O_3_ (111) Ti doped
Cu (100)	0.63	0.92	0.78	0.78	0.66	0.39
Cu (110)	0.49	0.81	0.84	0.72	−	−
Cu (111)	0.13	0.49	0.56	0.47	1.01	0.64
